# Patient factors affecting decision regret in the medical treatment process of gynecological diseases

**DOI:** 10.1186/s41687-019-0137-y

**Published:** 2019-07-17

**Authors:** Kiyomi Tanno, Seiji Bito

**Affiliations:** Division of Clinical Epidemiology, National Hospital Organization, Tokyo Medical Center, 2-5-1 Higashigaoka, Meguro-ku, Tokyo, 152-8602 Japan

**Keywords:** Decision-making, Japanese version of the decision regret scale, Latent class analysis, Multi-group analysis, Patient factors, Patient preference

## Abstract

**Background:**

To ensure that patients continue treatment, it is essential that the patient is satisfied with the decision-making process of the treatment. One way to address this is to assess the healthcare quality using the concept of regret, which can measure “Being convinced in decision-making.” This study aimed to elucidate patient factors affecting regret using the Japanese version of the Decision Regret Scale (DRS).

**Methods:**

A questionnaire survey was conducted with 197 patients with uterine myoma, ovarian tumors, and endometriosis. We then examined the relationship between the Japanese DRS, the Japanese SF-8 as a health-related quality of life (QOL), and patient factors using latent class analysis and path analysis through a multi-group comparison.

**Results:**

The final sample comprised 102 patients. Patients were classified into the following two groups based on the latent class analysis of patient characteristics: many patients who were married and had children and a few patients who were unmarried and had no children (class 1), and many patients who were unmarried and had no children and a few patients who were married and had children (class 2). The path analysis through the group comparison of the two classes revealed that subjective symptoms, preferences, and surgical procedure (laparotomy or laparoscopic surgery) had a direct impact on regret. The magnitude of the influence factors for Class 1 and Class 2 Regret was different. The indirect effect on regret was through mental component summary.

**Conclusion:**

Our results suggest that it is necessary to present treatment methods with consideration to patients’ backgrounds and to obtain informed consent from patients.

## Background

Since the second revision of the Medical Care Act in Japan in 1992, the government has aimed to create a system to provide high-quality and appropriate medical care. In the third revision of the Medical Care Act in 1998, a new target related to explaining the treatment procedure and obtaining the consent of patients (informed consent) was introduced. The promotion of treatment as an agreement between the patient and physician was also adopted as a goal.

In the process of making treatment decisions, it is essential that patients feel satisfied with their decisions. To evaluate the level of satisfaction with decision-making, it is extremely useful to assess the quality of treatment based on the concept of regret [1–3].

Previous studies in the field of psychology have investigated and demonstrated the relationships between decision-makers’ positivity and post-decision regret [4], decision-makers’ past experiences and differences in magnitude in post-decision regret [5], as well as the situation at the time of decision-making and differences in magnitude in post-decision regret [6].

A previous study showed that if a patient obtains undesirable results after treatment, the patient will regret the decision-making process [7]. Some evidence from cancer research suggests the importance of careful, well-informed patient decision-making. Based on this previous study, we expect that patients will recognize multiple subjective outcomes in a chain rather than recognizing multiple subjective outcomes simultaneously after treatment. The perception of one’s physical and mental QOL after treatment is thought to affect a patient’s satisfaction of pre-treatment decision-making.

In a previous study using the Japanese Decision Regret Scale (DRS), we verified the relationship between patient factors and regret [8]. This study was intended for patients with inguinal hernia, cholelithiasis, cholecystitis, or gallbladder polyps. The results showed that sex was a patient factor that directly influenced regret, with feelings of regret being significantly higher among men than among women. However, the patient factors used in this study were the patient’s background (age and sex), type of surgery, the presence or absence of complications, and did not include psychological factors of patients. Furthermore, over the last five years, studies using the DRS (on which the Japanese DRS is based) have tended to look at the relationship between processes and outcomes (e.g., health, HRQoL outcomes, and treatment outcomes) [9–11]. Sawka explored regret in thyroid cancer patients relating to the decision to accept or reject adjuvant radioactive iodine treatment [9]. This study revealed that thyroid cancer patients who reported that they were involved in the decision to engage in final adjuvant radioiodine treatment experienced less regret than those who did not. Zhong examined the regrets of decisions after breast reconstruction. Patients with low self-efficacy were dissatisfied with the information before surgery and revealed that they eventually regretted the decision to undergo breast reconstruction [10]. Chien targeted patients with localized prostate cancer in Taiwan. Education level, decision preferences, and psychosocial adjustment were associated with decisional conflict and influenced decision-making. The feeling of ineffective decision-making and decisional regret was low post-treatment. Psychosocial adjustment was associated with effective decision-making and decisional regret [11]. Therefore, we consider it necessary to investigate relationships with patient factors that influence outcomes, rather than to evaluate clinical decisions based solely on outcomes.

### The present study

**Fig. 1:**
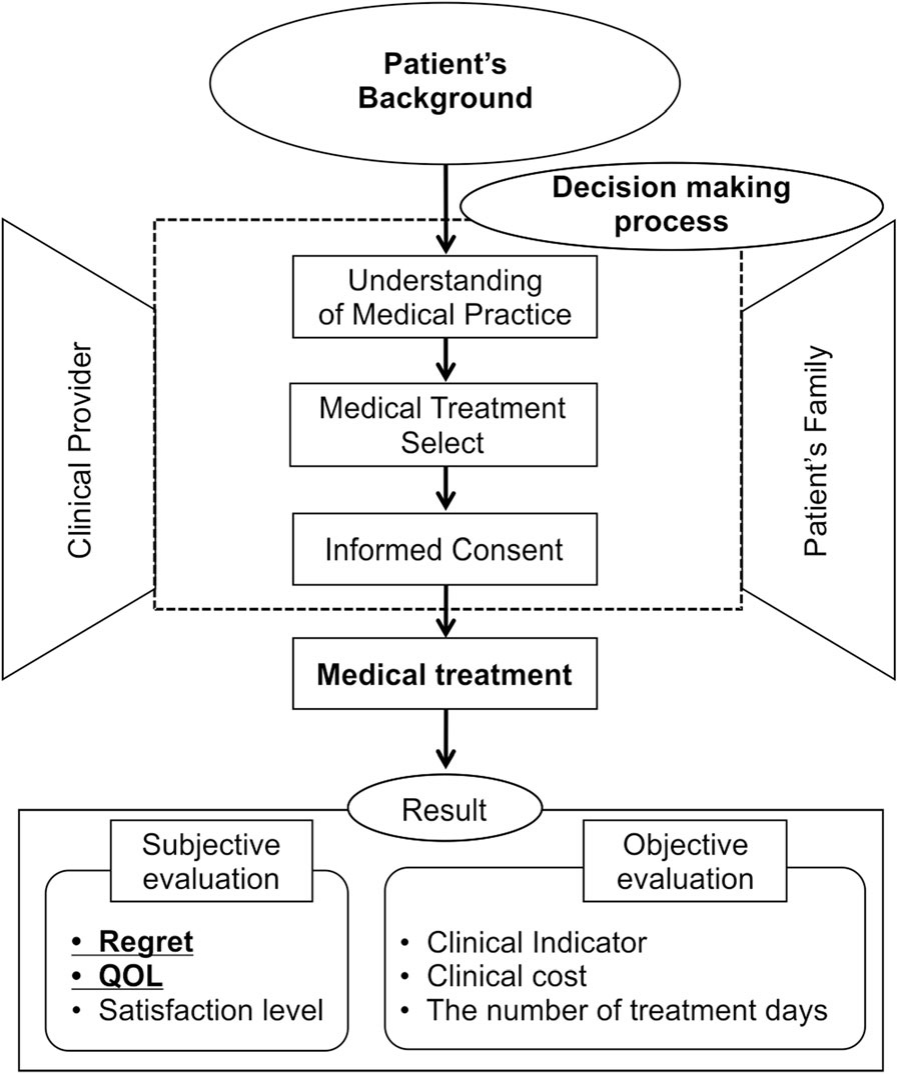
Conceptual diagram

A conceptual diagram concerning this study is shown in Fig. 1. The clinical process and influence factors from the disease occurrence to the result (after clinical treatment) are listed. As a result of this study, we chose Regret and QOL for subjective evaluation. This is because, in Japan, there is a scale for which validation of question items has been completed related to Regret and QOL. In this study, the Japanese version of the Japanese DRS [12] and health related QOL (SF-8) [13]) were used.

**Fig. 2:**
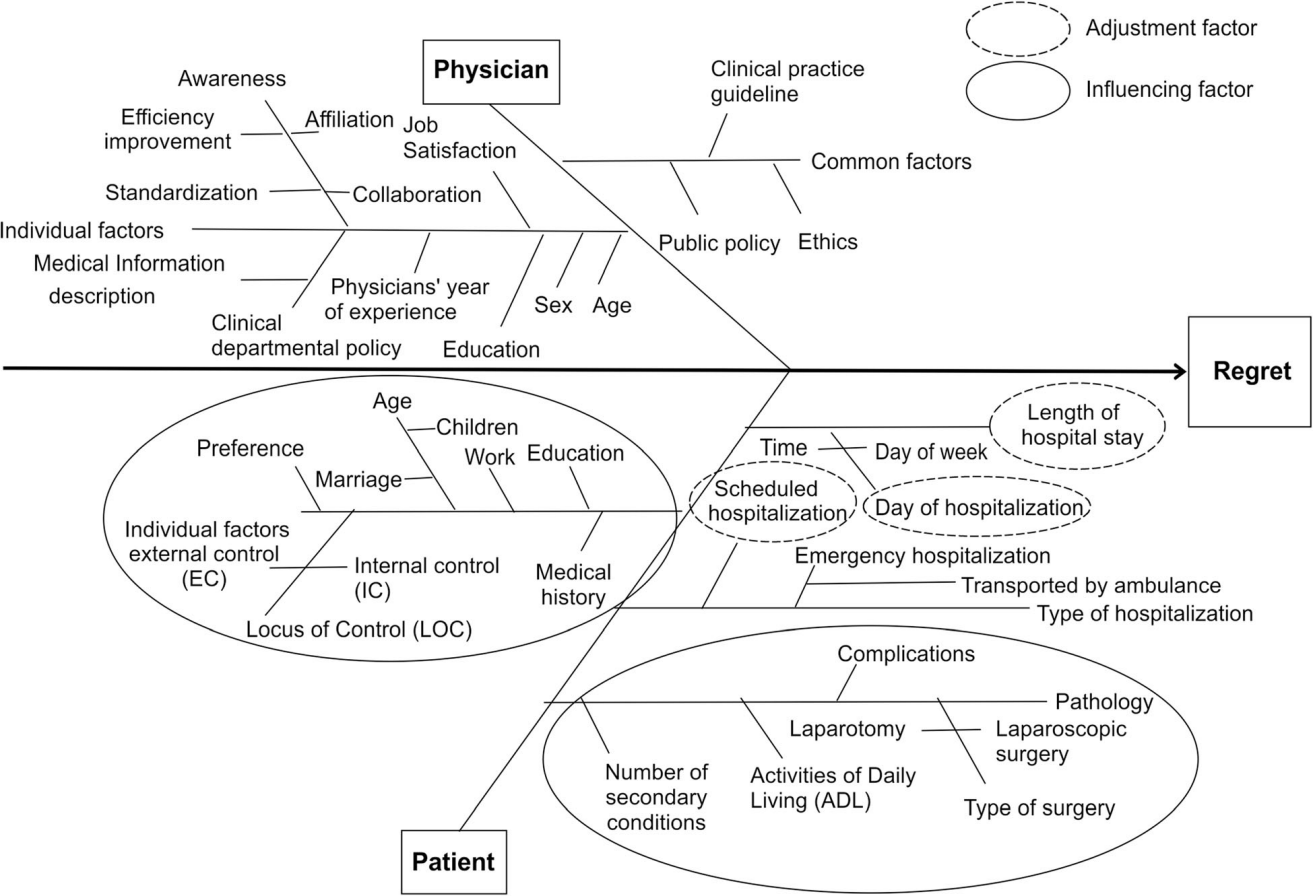
Fishbone chart

Figure 2 presents a fishbone chart of the characteristic factors that are considered to influence regret. We considered patient and patient family factors, physician factors, and medical personnel factors as likely to influence the clinical process. Patient factors included individual patient background [8, 14, 15], pathology [6], type of surgery [16], type of hospitalization, and the day of the week and time of hospitalization. For those with the subject conditions, we considered patients whose hospitalizations had been scheduled, excluding emergency hospitalizations, because, based on previous studies, consideration was given to differences in the allocation of medical resources according to type of hospitalization [17, 18] and the day of the week and time of hospitalization [19–22]. Further, scheduled hospitalizations were also thought to have been undertaken in conditions where the clinical process was formed through consensus between physicians and patients. Furthermore, the psychological characteristics of patients were also considered a factor influencing regret. The Locus of Control (LOC) scale was adopted to measure this psychological trait and was developed originally by Julian Lotter in the 1950s [23]. LOC refers to an individual’s perception of the underlying cause of an event (e.g., a disease) in a person’s life. For example, LOC can assess whether individuals believe their destiny is self-determined or dominated by an external force (e.g., destiny, God, or another powerful person). Internal LOC is seen as a trait of people who place their control of things within their personal abilities while external LOC is considered a trait where people place control of things outside of their own abilities. We created a questionnaire that reflects these aspects of LOC, which is described in the Methods section. Figure 2 shows a fishbone chart of the characteristic factors for the target disease. For other diseases (e.g., cancer), we would expect the factors to change. For example, in the case of a cancer patient, it is predicted that a disease state (stage) would be added to this fish bone.

In this study, we asked patients to evaluate their own decision-making, in the context of the clinical process, in terms of whether they were satisfied or harbored regrets once the clinical process was over. This subjective evaluation by the patients themselves constitutes an evaluation of the outcomes—that is, of decision making—in the clinical process. To assess “patient regret,” we used the Japanese version [12] of the Patient-Reported Outcomes DRS [24] developed by Brehaut et al., and to measure patients’ subjective outcomes, we used the Japanese SF-8 Health Survey (Japanese SF-8) [13] to estimate patient’s QOL. We focused on diseases affecting the female reproductive system, and thus restricted the sample to women.

The significance of this study is that, by verifying the relationship of QOL and patient factors to the Japanese DRS among patients suffering from female reproductive disorders, it will be possible to clarify the relationship of regret regarding decision-making with the subjective outcomes of patient preferences and patient characteristics, which are reflective of the diversity of contemporary women’s environments and backgrounds. Also, this review could clarify the relationship between regret about decision-making regarding treatment of gynecological reproductive disorders, subjective outcomes of patient preferences, and patient characteristics. In addition, we believe that it would be possible to propose medical interventions based on this empirical evidence, and identify how best to provide appropriate information based on different patient characteristics. Additionally, these results could help establish a forecasting model that can be used to develop future decision-making guidelines. In addition, we believe it could be possible to provide care that patients view as efficient and appropriate in the future. In Japan, while there is substantial research on the structure of patients’ decision-making up to the point of deciding to undergo surgery for breast cancer [25] and the process of selecting surgical procedures [26], research on decision-making by patients suffering from female reproductive disorders is comparatively rare. Therefore, we believe that the present study holds significance.

In this study, we set the following hypotheses:

1. Patient factors that influence regret would differ according to patient background.
2. Patients’ preferences would influence their regret.
3. Indirect effects that were mediated by QOL from patient characteristics to patient regret would be greater than the direct effect from patient characteristics to patient regret.

## Methods

### Design and setting

We conducted a retrospective cohort study in a hospital designated as a tertiary emergency medical facility in a metropolitan area with 780 patient beds and 34 clinical departments. We utilized a questionnaire survey with patients and extracted patient information.

### Participants

We targeted patients whose background information was stored in the MEDI-ARROWS Diagnosis Procedure Combination (DPC) analysis system in the study hospital and who satisfied the eligibility criteria. Among patients discharged between August 2012 and July 2013 with no psychiatric medical history and not currently receiving psychiatric treatment, eligibility criteria were met by 209 individuals, including 119 individuals who underwent surgery for myomas (DPC code 120060), 88 individuals who underwent surgery for benign ovarian tumors (DPC code 120070), and 2 individuals who underwent surgery for endometriosis (DPC code 120100). Consideration was given to the volume of medical resources allocated and all were regarded as patients who were scheduled for hospitalization, excluding emergency hospitalizations.

### Questionnaire survey

For the survey, a questionnaire was distributed and collected by post. The survey period lasted from September 25 to October 10, 2014. We attempted to collect data while the patients’ recollections were clear, so the patients had been discharged for 1–2 years before the survey. In addition, the differences in length of time since discharge between patients was no more than one year, so fluctuations in patient memory were small. The questionnaire survey did not prompt the participants to return it after it was mailed. The response rate was the result of only one mailing.

The questionnaire survey used in this study was prepared using items from three evaluation scales, namely 5 from the Japanese DRS, 8 from the Japanese SF-8 Health-Related QOL scale, and 18 from the Locus of Control (LOC) Scale of behavioral change, used as one of the patient factors. Thus, a total of 31 items was used.

For the use of the Japanese SF-8 and LOC scale, we obtained permission from their respective copyright holders. The Japanese SF-8 [13] is a self-report health-related QOL scale based on eight areas (physical functioning, role physical, bodily pain, general health perception, vitality, social functioning, role emotional, and mental health). Eight questions are answered using a 5 or 6-point Likert scale. In this study, we used two aggregate scales, physical component summary (PCS) and mental component summary (MCS), calculated from each subarea. The Japanese version of the LOC Scale, developed by Kanbara et al. [27], comprises 18 items (10 on internal control and 8 on external control), each of which is graded on a 4-point scale. The internal control items are scored from 4 points for “Agree” to 1 point for “Disagree,” and the scoring scheme is reversed for the external control items. The higher the score, the stronger the tendency toward internal control.

### Extraction of patient information

We extracted patients’ background, treatment, pathology, and behavioral factors from electronic medical records. Table 1 shows the variables and levels (factors and categories). It should also be noted that the LOC groupings for patient background factors were obtained from responses to the questionnaire. The top 35% and bottom 35% of LOC scores were extracted and classified as High and Low, respectively, and all others were classified as Middle [27].

**Table 1:** Factors and categories

Factor	Categories	
Age (years)	< 29	
	30 to 39	
	40 to 49	
	50 to 59	
	> 60	
Married	Not married	
	Married	
Children	Have no children	
	Have children	
LOC group	Low	
	Middle	
	High	
Surgical procedure (1)	0:	Enucleation
	1:	Total removal
Surgical procedure (2)	0:	Laparotomy
	1:	Laparoscopic surgery
Subjective symptoms	0:	Not present
	1:	Present
Cancer history	0:	Not present
	1:	Present
Illness after discharge	0:	Not present
	1:	Present
Preference	0:	Not present
	1:	Present
Key person	0:	Not present
	1:	Present

*LOC* locus of control, *Low* the bottom 35% of LOC scores, *Middle* LOC scores not classified as Low or High, High, the top 35% of LOC score

Cancer history refers to the presence or absence of experience with cancer in other parts of the body prior to acquiring the target disease. A patient was defined as having a preference when the facts listed in their electronic medical records indicated a desired time of surgery, desired treatment policy, implementation of a second opinion, or receipt of counseling from a medical social worker.

### Ethical considerations

This study was carried out with the approval of the Ethical Review Committee of the hospital where the study was conducted. Respondents were instructed, in writing, about the purpose of the survey and were informed that their participation was voluntary, they would suffer no disadvantage if they declined participation, and their anonymity would be secure. Completion of the consent form sent along with the questionnaire was regarded as consent to participate. Questionnaires were unsigned. Furthermore, because patients’ information was extracted from the electronic medical records, the data used in the analysis did not include information such as patient ID, patient names, or patient addresses, which would make it possible to identify individual patients.

### Analytical methods

We verified our hypotheses using a two-stage analytical method. In the first stage, we performed a latent class analysis to classify the data according to patient background. For this, we used log-linear and event history analysis with missing data using the Expectation Maximization (EM) algorithm [28]. The number of latent classes was selected using Bayesian Information Criterion (BIC) to test the goodness-of-fit of the model [29, 30].

In the second stage of the analysis, we performed a path analysis through structural equation modeling using IBM SPSS Amos Graphics 22 (IBM Japan, Tokyo). In the path analysis, we conducted a mean structure analysis for a multi-group population for each of the classes identified by the latent class analysis in the first stage, and then repeated modeling improvements. For judging the goodness-of-fit of the model, we used the comparative fit index and root mean square error of approximation (RMSEA), judging the model to conform to the data when the CFI was at least 0.80 and RMSEA was less than or equal to 0.05. We also selected the optimal model using Akaike information criterion, which is an indicator of a model’s relative goodness-of-fit [31].

**Fig. 3:**
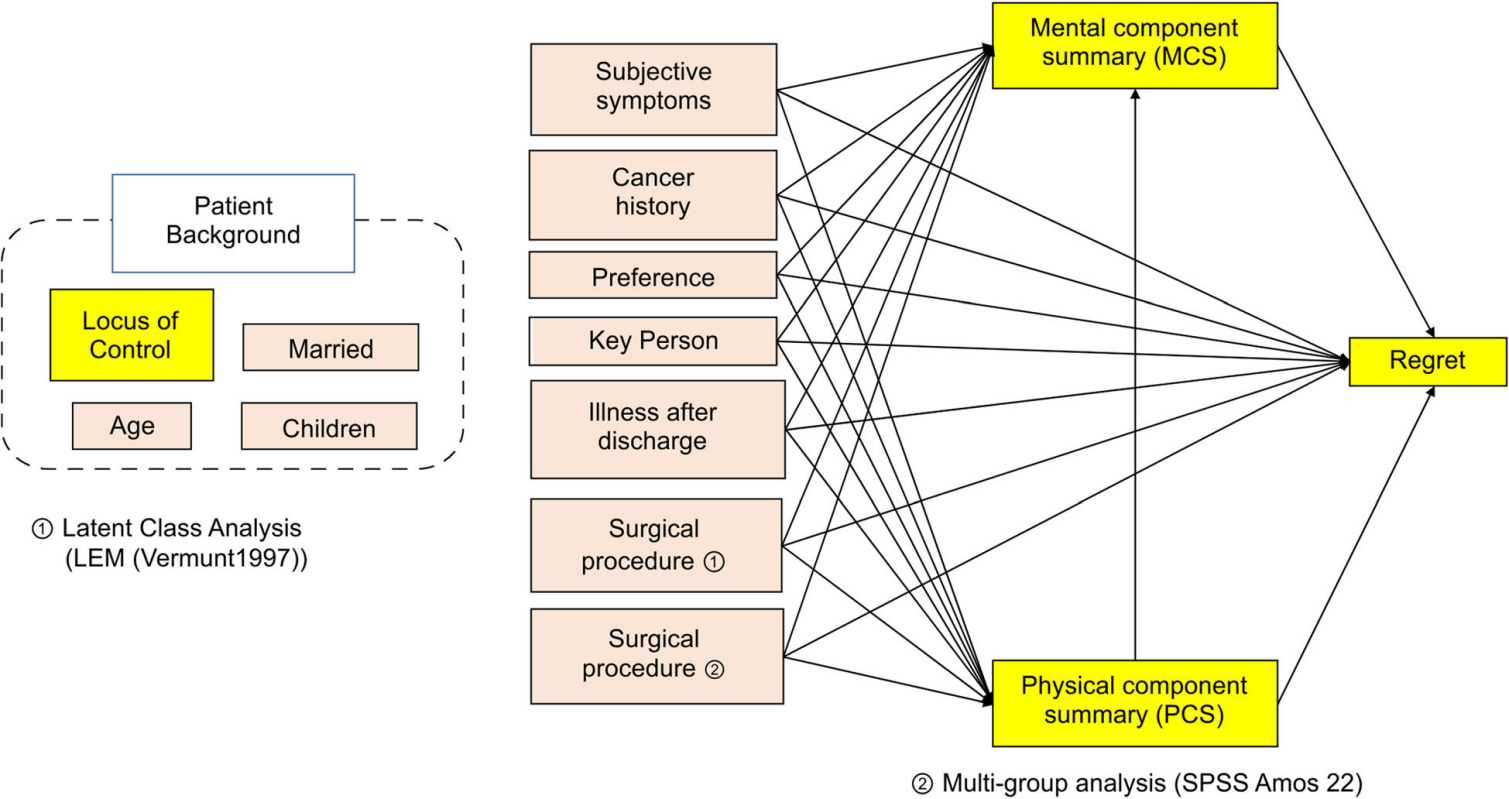
Analytical method. LEM, log-linear and event history analysis with missing data using the EM algorithm. This path diagram was created based on the study hypotheses, and the path analysis was performed based on this path diagram

Figure 3 shows a path diagram of the hypotheses mentioned earlier. The underlined factors and outcomes were obtained from the questionnaire survey, while other factors were obtained from the electronic medical records. For outcomes, we considered the two summary PCS and MCS scores of the SF-8 scale, which measure health-related QOL, and regret on the Japanese DRS. Regarding the outcome scores, higher PCS and MCS scores indicate a better sense of well-being, while a higher regret score shows a stronger sense of regret.

To verify Hypotheses 1 and 2, we set a path to regret, from the information for each patient. To verify Hypothesis 3, we set a path to regret via PCS and MCS, from the information for each patient. The model constituted a fully sequential model.

The funding agreement ensured the authors’ independence in designing the study, interpreting the data, and writing and publishing the report.

## Results

### Respondent attributes

After excluding patients whose address was unknown or who did not meet the eligibility criteria, we sent out 197 copies of the self-administered questionnaire survey, of which 104 were collected (recovery rate of 53%). The number of valid responses was 102, which was regarded as the target sample for the analysis. The mean age of respondents was 47 years old (SD 10, range 28–82), which was over middle age from the characteristics of the disease was high rate of morbidity over middle age. The mean Japanese DRS score was 10.28 (SD 12.01, range 0–50), which was lower than the mean of 16.50 (*n* = 44 test) from a systematic review of the DRS [32]. Forty-four studies reported mean scores ranging from 2.5 to 49.1 out of 100, with an overall across-studies mean score of 16.5 and a median of mean scores of 14.3 The mean SF-8 (PCS) score was 50.66 ± 5.13 (range 34.99–58.38), and the mean SF-8 (MCS) score was 49.88 ± 5.59 (range 29.70–56.37). Compared with the Japanese national standard value of 50 [13], scores in this sample for both PCS and MCS were similar.

### Results of the latent class analysis (patient background classification)

The basic aggregations (frequency distribution) of each of the patient background factors and the summarized statistical results of each model are shown in Table 2. From the goodness-of-fit of each class, the determination of the number of classifications based on BIC yielded a 2-class solution. The lower the BIC value, the better the model.

**Table 2:** Goodness-of-fit of each class

Class	L^2^	df	p-value	BIC
1	96.166	51	0.000	795.163
2	**37.374**	**46**	**0.814**	**759.546**
3	32.910	41	0.812	778.207
4	27.735	36	0.836	796.156
5	20.780	31	0.917	812.326

*L*^*2*^ Likelihood ratio chi-square statistic, *df* degrees of freedom, *BIC* Bayesian information criterion. The lower the BIC value, the better the model is set in bold, so we decided on two classes

Table 3 shows the constitution ratio of each latent class in the two-class model that was adopted, that is, “many patients who were married and had children and a few patients who were unmarried and had no children” (Class 1) or “many patients who were unmarried and had  no children and a few patients who were married and had children” (Class 2). “Age” for both classes was “under 40 years,” and while no striking differences were apparent, for the “LOC groups,” Class 1 exhibited more internal control tendencies, while external control tendencies were more or less comparable in both classes.

**Table 3:** Composition ratio of each latent class and the conditional response probability in the 2-class model

Variable	Category	Class 1	Class 2
Class Size		0.563	0.437
Married	Not Married	0.072	**0.782**
	Married	**0.928**	0.218
Children	Have no children	0.097	**0.953**
	Have children	**0.903**	0.047
Age (Years)	< 29	0.158	**0.403**
	30 to 39	**0.429**	**0.457**
	40 to 49	0.284	0.127
	50 to 59	0.030	0.006
	> 60	0.098	0.008
LOC	Low	0.271	**0.392**
	Middle	0.242	0.249
	High	**0.488**	**0.359**

Class 1: Many patients who were married and had children and a few patients who were unmarried and had no children

Class 2: Many patients who were unmarried and had no children and a few patients who were married and had children

LOC, Locus of Control

Highlighted about 0.4 or more is set in bold so that the composition of each class can be judged

Furthermore, Table 4 shows a comparison of the Japanese DRS for the two classes. The score for Class 1 was 11.16 ± 12.39, while that for Class 2 was 9.21 ± 11.57. While there was no significant difference, scores were higher in the former group, and variance was also quite high.

**Table 4:** Descriptive statistics of the Japanese DRS of 2 classes and the result of the t-test

Class	N	Minimum	Maximum	Mean	SD	t-value	*p*-value
Class 1: Many patients who were married and had children and a few patients who were unmarried and had no children	56	0.00	50.00	11.16	12.39	0.81	0.418
Class 2: Many patients who were unmarried and had no children and a few patients who were married and had children	46	0.00	35.00	9.21	11.57		

*Japanese DRS* = Japanese version of the Decision Regret Scale

### Results of the path analysis

To verify the difference in the effect of factors on regret based on the two-class patient background characteristics identified in the latent class analysis, we conducted a multi-group comparison using path analysis. To verify the heterogeneity of “Class 1: many patients who were married and had children and a few patients who were unmarried and had no children” and “Class 2: many patients who were unmarried and had no children and a few patients who were married and had children,” we first carried out the path analysis using the constructed models in Fig. 3, and then searched for the most appropriate model in terms of goodness-of-fit. Here, the whole model had a χ^2^ value of 1.568 and a *p*-value of 0.980. The CFI was 1.000 and RMSEA was 0.000, indicating good fit.

**Fig. 4:**
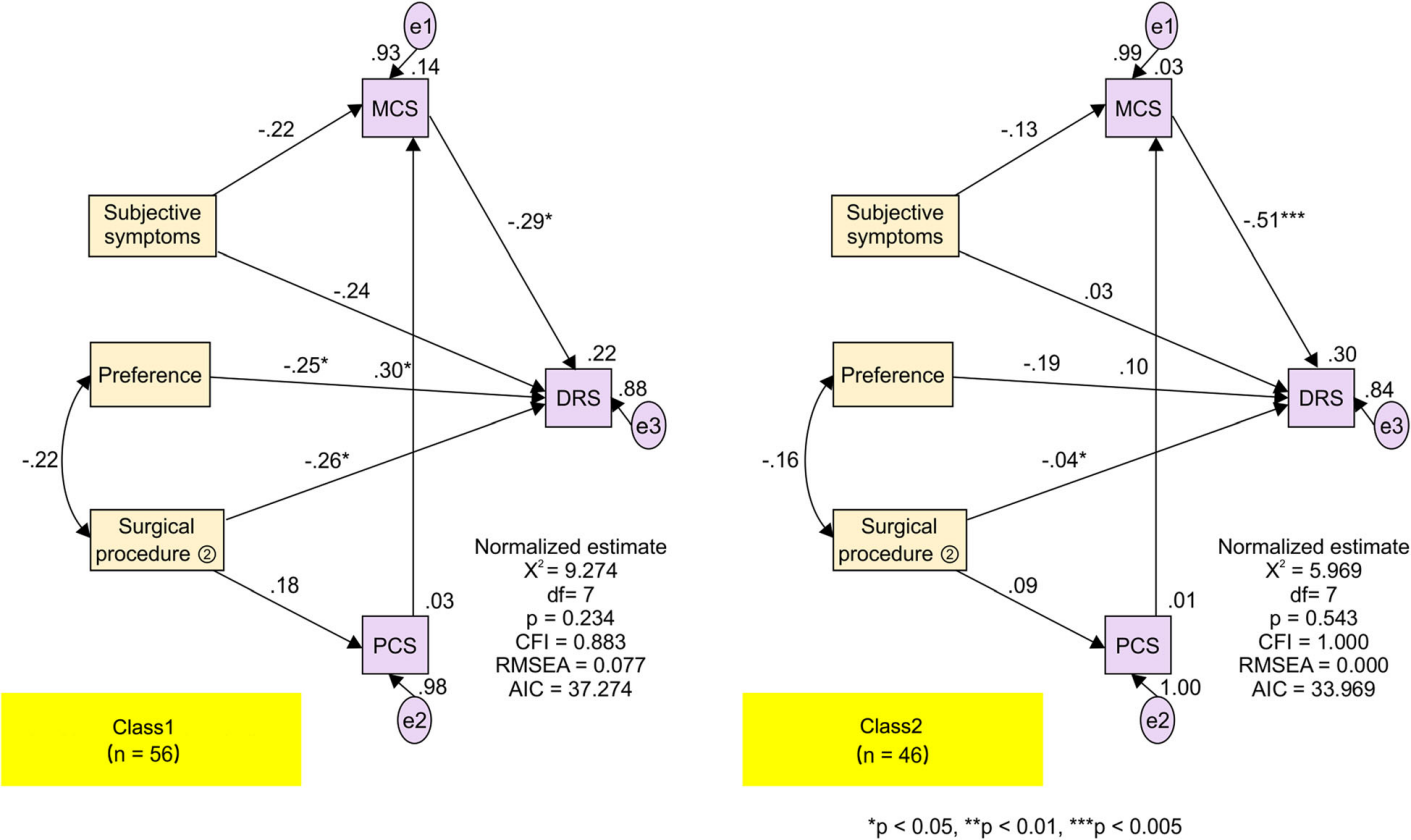
Multi-Group Analysis. Subjective symptoms (0 Not present, 1 present), Preference (0 Not present, 1 present), and Surgical procedure (2) (0 laparotomy, 1 laparoscopic surgery). MCS, Mental component summary; PCS, Physical component summary; DRS, Japanese version of the Decision Regret Scale; df, degrees of freedom; CFI, comparative fit index; RMSEA, root mean square error of approximation; AIC, Akaike information criterion

When the path analysis for each class was carried out using the whole model, Class 1 had a χ^2^ value of 9.274 and a p-value of 0.234, with conformity indexes of 0.883 for CFI and 0.077 for RMSEA, while Class 2 had a χ^2^ value of 5.969 and a p-value of 0.543, with conformity indexes of 1.000 for CFI and 0.000 for RMSEA, indicating goodness-of-fit in both cases. These results are shown in Fig. 4.

From the above results, we considered that it was not unreasonable to analyze the two classes using the same model, and therefore, we proceeded to confirm configural invariance. We performed a confirmation to evaluate the hypothesis that, with no equality constraints due to configural invariance, the measured values may be different from each other even for the same path diagram between the two classes. Good results were obtained for the conformity indexes in this analysis, CFI of 0.958 and RMSEA of 0.030, indicating that configural invariance was established.

Because configural invariance was confirmed, we also investigated a model with equality constraints between the classes. Here, the conformity indexes yielded a CFI of 0.839 and RMSEA of 0.053, and it was confirmed that the fit of the model was better without constraints. Furthermore, for Akaike information criterion, as well, no constraints yielded 71.239 and constraints yielded 71.696. It was judged that the fit of the model was better without constraints, though only slightly so. Comparisons were thus made for the measured values for each class without the imposition of equality constraints.

Figure 4 shows the results of the multi-group comparison for each class. Subjective symptoms, preference, and surgical procedure (2) (either laparotomy or laparoscopic surgery) were found to have a direct influence on regret.

Subjective symptoms also had paths to MCS and to regret. In Class 1, those with subjective symptoms had low MCS (− 0.22) and low regret (− 0.24). Those in Class 2 with subjective symptoms also had low MCS (− 0.13), though it was suggested that their regret was slightly higher (0.03). In an interclass comparison, the path coefficient for Class 1 was larger, demonstrating a strong influence.

Preference had a path solely to regret, and it was suggested that, in both Class 1 and Class 2, regret scores would be lower in the higher presence of “preference” (Class 1 = − 0.25, Class 2 = − 0.19). In an interclass comparison, the path coefficient for Class 1 was found to have a highly significant path, demonstrating a strong influence.

Surgical procedure (2) had paths to PCS and regret. In both Class 1 and Class 2, patients who underwent laparoscopic surgery had higher PCS and lower regret scores than did patients who underwent laparotomies (Class 1 = − 0.26, Class 2 = − 0.04). In an interclass comparison, the path coefficient for Class 1 was larger, and the path coefficient to regret was particularly significant, demonstrating a strong influence.

In terms of the influence of the SF-8 (PCS and MCS), PCS had no direct path to regret. In both Class 1 and Class 2, it was suggested that MCS was higher when PCS was higher (Class 1 = 0.30, Class 2 = 0.10). The path coefficient from PCS to MCS was larger in Class 1, showing a strong influence.

MCS had a direct influence on regret, and in both Class 1 and Class 2, it was suggested that as MCS rose, regret scores decreased (Class 1 = − 0.29, Class 2 = − 0.51). This was a significant path for both classes, with a particularly strong influence in Class 2.

## Discussion

### The influence of patient factors on regret

In the latent class analysis, we were able to isolate two classes of patients, namely Class 1 and Class 2. A test of the difference of the mean scores for each class on the Japanese DRS (Table 4) showed no significant difference. The absence of any difference was thought to indicate that marriage, children, age, and LOC had no influence on regret, and that differences in the influence of patient factors on regret by patient background could be comparatively examined in each class.

Subjective symptoms affected regret more strongly in Class 2 than in Class 1. In addition, the signs of Class 1 and Class 2 path coefficients were reversed, which is a unique result. Loewenstein investigated and demonstrated that regret was larger when decisions were urgently required than when decisions could be made with caution [6]. With regard to differences in the magnitude of post-decision-making regret and the situation at the time of decision-making, it has been reported that individuals in a “hot” condition—for example, feeling strong dissatisfaction, hunger, or pain—are less likely to exercise caution when making choices than when they are in a calm or “cold” condition. The relationship between “subjective symptoms” and regret among Class 2 seemed to be a weaker form of the same result. In addition, in terms of patient background, because this class included younger patients than those in Class 1, it was also expected that they would be thinking about getting married and having children in the future, and so they tended to be more cautious when evaluating treatment options. On the other hand, Class 1 exhibited a negative relationship between regret and “subjective symptoms.” In this case, regret is low due to the disappearance of “subjective symptoms,” that is, they are satisfied with their decision-making. This was also suggested by the fact that the influence of “subjective symptoms” on MCS was negative, and that this was stronger for Class 1 than it was for Class 2.

The presence or absence of “preference” had a direct effect on regret: regret scores were lower among patients who expressed their “preference.” Similar to findings of a study that showed neither knowledge nor intervention, but rather harmony between preference and decision is necessary for engaging in informed choices in the context of making treatment decisions about myomas [33], the present finding seems to clarify the necessity of “preference.” In addition, based on a report that revealed that, owing to the variation in medical costs in the context of myomas (the amount of medical resources allocated), medical treatment for benign tumors is based on patient choice, it became clear from patients’ own evaluations that satisfaction was achieved through the expression of “preference” [34]. In the present study, the influence of preference was larger among Class 1 than it was among Class 2. In this case, it was thought that the female-specific background of the patients, including situations where patients also need to care others (e.g., getting married, having children) was expressed through such preference.

“Surgical procedure (2)” affected both PCS and regret. While PCS was higher and regret scores were lower for laparoscopic surgery than for laparotomies, in both cases the impact was larger on Class 1 than on Class 2. In particular, the impact on regret was significantly large for Class 1. It was suggested that low invasiveness, high economic efficiency, cosmetic appeal [35], postoperative pain relief, and reduction of the length of hospitalization [36] were associated with laparoscopic surgery, which was particularly suited to the needs of women in Class 1.

Furthermore, a negative correlation was found to exist between the factors of “preference” and “surgical procedure (2).” It was found that those who did not express a “preference” underwent laparoscopic surgery for “surgical procedure (2).” This result seems to reflect the fact that, since all benign tumors in gynecological areas were cases for laparoscopic surgery, this approach was applied except in cases where a laparotomy was particularly necessary. Moreover, it seems to reflect that laparoscopic surgery served patients’ needs.

### The process of recognizing regret (direct and indirect effects)

Subjective symptoms, preference, and surgical procedure (2) were all found to exert a direct influence on regret. The selection of medical treatment according to the will of the patient, as indicated in a report that found harmony between preference and decision [33], also seems to require harmony between individual factors. In addition, it became clear that all factors had a stronger influence on female participants in Class 1 than on those in Class 2. Because the impact of patient background, and especially of patients’ families, may have a major influence, physicians should present patients with information about factors that directly influence regret, as well as take patients’ backgrounds into account when discussing the timing and method of treatment.

It was also found that subjective symptoms exerted an indirect effect on regret via MCS, and that surgical procedure (2) exerted an influence on regret through MCS from PCS. We posited a hypothesis that, in cases of undesirable results after treatment, the clinical process prior to decision-making could sometimes also lead to regret [7] as an indirect effect of the awareness of the results. This hypothesis was proven for subjective symptoms and surgical procedure (2). In particular, the effect from PCS to MCS was larger for Class 1 than it was for Class 2. In Class 1, direct effects and indirect effects were of an equivalent size. A class-based difference was suggested in that the influence from MCS on regret was larger in Class 2 than in Class 1. Furthermore, it was predicted that Class 2 would include more working women, among whom indirect effects on regret would be felt more strongly than direct effects. It seems that the influence of patients’ emotional and social sense of well-being has a larger influence on regret than does physical sense of well-being.

In this study, it became clear that there are two patterns: 1) patient factors that directly affect remorse, and 2) indirect patient factors that affect regret after having noticed QOl. We have described both the direct and indirect effects on the regret. Overall, such verification is rare. Even in recent research [37–40], there has been no decision-making regret research that has taken the patient’s context into consideration nor has any study shown that patient factors affecting regret differ depending on the patient’s background. Thus, the current findings extend the existing field of study regarding regret and medical decision-making.

### Limitations

As this study involved the consideration of a single facility, its reflection of the lifestyle of women may be restricted to those living in a particular geographical area. It will be necessary to widen the area to verify the same hypotheses while also considering regional and cultural differences.

In addition, results obtained from the path analysis in this study were the primary focus, and we did not consider interactions. While it is necessary to confirm interactions, the large number of factors made it difficult to carry out an analysis to supplement the factors with interaction items, and it was also difficult to confirm the combination of each item by performing a two-way analysis of variance. Therefore, we decided to avoid interactions completely in the classification by patient background that was conducted in the first stage of analysis.

Furthermore, with regard to patient factors, the effects of bias may be of concern, as patient preferences were extracted from the patients’ electronic records.

## Conclusions

This study has clarified several points. First, while “subjective symptoms,” “preference,” and “surgical procedure (2)” all exerted an influence on regret, it was found that this influence was stronger among women in Class 1 than it was among women in Class 2. Second, expression of “preference” in the clinical treatment process was found to exert an influence on regret. Third, while regret among women in Class 1 arose as both a direct and indirect effect of patient characteristics, it was found that regret among women in Class 2 was more likely to be felt as an indirect effect than as a direct effect of patient characteristics, and that regret was particularly subject to the influence of emotional and social sense of well-being.

In this study, aside from patient factors directly influencing regret, differences also became clear in the effect of patient factors on regret according to patient background. Thus, it is suggested that physicians should listen to patient preferences regarding details such as treatment time and method. They should also consider patients’ background in terms of their family and work situation, as well as with reference to conditions and treatment methods that have a direct impact on possible future regret.

## Data Availability

The data collected during this study is unavailable to the public due to confidentiality concerns. Reasonable requests to review the data for scientific and/or research purposes may be considered with permission of the National Hospital Organization, Tokyo Medical Center.

## Abbreviations

BIC: Bayesian information criterion
DPC: Diagnosis procedure combination
DRS: Decision Regret Scale
LOC: Locus of control
MCS: Mental component summary
PCS: Physical component summary
QOL: Quality of life
RMSEA: Root mean square error of approximation

## References

[1] Bell DE (1982). Regret in decision making under uncertainty. Operations Research.

[2] Kahneman D, Miller DT (1986). Norm theory: Comparing reality to its alternatives. Psychological Review.

[3] Zeelenberg M, Beattie J, van der Pligt J, de Vries NK (1996). Consequences of regret aversion: Effects of expected feedback on risky decision making. Organizational Behavior and Human Decision Processes.

[4] Tversky A, Kahneman D (1981). The framing of decisions and the psychology of choice. Science.

[5] Zeelenberg M, van Dijk WW, van der Pligt J, Manstead ASR, van Empelen P, Reinderman D (1998). Emotional reactions to the outcomes of decisions: The role of counterfactual thought in the experience of regret and disappointment. Organizational Behavior and Human Decision Processes.

[6] Loewenstein G (2005). Hot-cold empathy gaps and medical decision making. Health Psychology.

[7] Connolly T, Reb J (2005). Regret in cancer-related decisions. Health Psychology.

[8] Tanno K, Takagi Y (2015). Relationships among the score on a Japanese version of the “decision regret scale,” score on the health-related quality of life scale, and patient factors- a cross-sectional study in patients with inguinal hernia, cholelithiasis, cholecystitis, and gallbladder polyp. Journal Japanese Society Healthcare Administrative.

[9] Sawka AM, Straus S, Gafni A (2012). Thyroid cancer patients’ involvement in adjuvant radioactive iodine treatment decision-making and decision regret: An exploratory study. Supportive Care in Cancer.

[10] Zhong T, Hu J, Bagher S, O’Neill AC, Beber B, Hofer SO, Metcalfe KA (2013). Decision regret following breast reconstruction: The role of self-efficacy and satisfaction with information in the preoperative period. Plastic and Reconstructive Surgery.

[11] Chien CH, Chuang CK, Liu KL, Li CL, Liu HE (2014). Changes in decisional conflict and decisional regret in patients with localised prostate cancer. Journal of Clinical Nursing.

[12] Tanno K, Bito S, Isobe Y, Takagi Y (2016). Validation of a Japanese version of the decision regret scale. Journal of Nursing Measurement.

[13] Fukushima S, Suzukamo Y (2004). Manual of the SF-8 Japanese version.

[14] Eagly AH, Carli LL (1981). Sex of researchers and sex-typed communications as determinants of sex differences in influenceability: A meta-analysis of social influence studies. Psychological Bulletin.

[15] Reker GT, Peacock EJ, Wong PT (1987). Meaning and purpose in life and well-being: A life-span perspective. Journal of Gerontology.

[16] Ido K, Kimura K, Kawamoto C (1991). Laparoscopic laser cholecystectomy: A prospective analysis of 70 initial cases. Nihon Shokakibyo Gakkai Zasshi.

[17] Fujimura H (2010). Evaluation of the diagnosis procedure combination payment system. Japanese Journal Political Economy Health Health Care.

[18] Sechiyama Y (2010). Necessity of comprehensive evaluation by diagnosis procedure combination (DPC) that distinguishes scheduled/emergency hospitalization. Japanese Journal Hospital Association.

[19] Arias Y, Taylor DS, Marcin JP (2004). Association between evening admissions and higher mortality rates in the pediatric intensive care unit. Pediatrics.

[20] Bell CM, Redelmeier DA (2001). Mortality among patients admitted to hospitals on weekends as compared with weekdays. The New England Journal of Medicine.

[21] Jneid H, Fonarow GC, Cannon CP (2008). Impact of time of presentation on the care and outcomes of acute myocardial infarction. Circulation.

[22] Kostis WJ, Demissie K, Marcella SW, Shao Y-H, Wilson AC, Moreyra AE, Study Group (2007). Weekend versus weekday admission and mortality from myocardial infarction. The New England Journal of Medicine.

[23] Rotter J (1966). Generalized expectancies for internal versus external control of reinforcements. Psychological Monographs.

[24] Brehaut JC, O’Connor AM, Wood TJ, Hack TF, Siminoff L, Gordon E, Feldman-Stewart D (2003). Validation of a decision regret scale. Medical Decision Making.

[25] Onuma N, Kamakura Y, Hasegawa M (2004). The structure of decision-making with regard to surgical treatment in breast cancer patients. J Jap Soc Nurs Res.

[26] Kokufu H, Inoue T (2002). The process of decision-making surgical treatment by breast regarding cancer patients. Japan Journal of Nursing Science.

[27] Kambara M, Higuchi K, Shimizu N (1982). Creation of locus of control scale, verification of reliability and validity. Japanese Journal of Educational Psychology.

[28] Vermunt, J. K. (1997). IEM: A general program for the analysis of categorical data. Tilburg University: Department of Methodology and Statistics.

[29] Miwa T (2009). Introduction to latent class model. Socio Theory Methods.

[30] Fujihara S, Ito T, Tanioka K (2012). Quantitative sociological approaches using the latent class analysis: Data analysis of status inconsistency, attitudes to social inequality, and authoritarian-conservatism. Annal Hum Sci.

[31] Toyoda H (2007). Covariance structure analysis (Amos version).

[32] Becerra Pérez MM, Menear M, Brehaut JC, Légaré F (2016). Extent and predictors of decision regret about health care decisions: A systematic review. Medical Decision Making.

[33] Solberg LI, Asche SE, Sepucha K (2010). Informed choice assistance for women making uterine fibroid treatment decisions: A practical clinical trial. Medical Decision Making.

[34] Tanno K, Bito S, Takagi Y (2014). Research on how physicians' awareness result in medical service fee variation: The analysis based on diagnosis procedure combination data. Jap J Qual Safe Health.

[35] Akira S, Araki T (2000). Endoscopic surgery in obstetrics and gynecology. J Jap Surg Soc.

[36] Suginami H (1998). Laparoscopic surgery in gynecology. Japanese Journal National Medicine Services.

[37] Wilkie DD, Solari A, Nicholas R (2019). Initiating disease-modifying treatments in multiple sclerosis: Measuring the decision process using decisional conflict and decisional regret scales. Multnomah Scler Journal Experimental Translational Clinic.

[38] Advani Pragati G., Lei Xiudong, Swanick Cameron W., Xu Ying, Shen Yu, Goodwin Nathan A., Smith Grace L., Giordano Sharon H., Hunt Kelly K., Jagsi Reshma, Smith Benjamin D. (2019). Local Therapy Decisional Regret in Older Women With Breast Cancer: A Population-Based Study. International Journal of Radiation Oncology*Biology*Physics.

[39] Wee Christina C., Fleishman Aaron, McCarthy Ashley C., Hess Donald T., Apovian Caroline, Jones Daniel B. (2019). Decision Regret up to 4 Years After Gastric Bypass and Gastric Banding. Obesity Surgery.

[40] Liu KL, Chien CH, Hsieh CY, Huang XY, Wang HH, Lin KJ, Chiang YJ (2018). Effective decision-making and decisional regret in living kidney donors of Taiwan. Transplantation Proceedings.

